# Editorial: Women in renal pharmacology: 2021

**DOI:** 10.3389/fphar.2022.1054354

**Published:** 2022-10-21

**Authors:** Ariela Benigni, Susanna Tomasoni

**Affiliations:** Istituto di Ricerche Farmacologiche Mario Negri IRCCS, Bergamo, Italy

**Keywords:** sex, chronic kidney disease, diabetic nephropathy, angiotensin II receptor blocker, receptor tyrosine kinases

There remains a considerable lack of knowledge about the impact of sex on human physiology and pathophysiology. This is in part because most pre-clinical research and drug development studies have been performed using male animal models, inserting a bias at the origins of the experimental design. It has become clear in recent years that sex differences play a fundamental role in most diseases, including chronic kidney disease (CKD), and women–whose biological susceptibility to a disease is different to that of men–may respond differently to therapy.

In the context of renal diseases, chronic kidney disease is more prevalent among women. Indeed, while CKD is the ninth leading cause of death for women, it is not included among the ten leading causes of death for men (Lancet 2020; 396: 565–82). However, the prevalence of end stage renal failure is higher in men because of a combination of physiological factors and risk factors, such as hypertension and obesity, which independently modulate the progression of renal disease (Semin Nephrol 2022; 42:101–113).

This concept has been highlighted and discussed within this Research Topic in the clinical study presented by Perna and collaborators. In this study the authors investigated whether and to what extent a patient’s response to Rituximab—one of first-line therapies for patients with membranous nephropathy (MN) who are at high risk of progression towards kidney failure—could be affected by both sex and anti-M-type phospholipase A2 receptor (antiPLA2R) antibody levels in a large and well-defined cohort of consecutive patients with primary MN and persistent nephrotic syndrome. Their in-depth analysis shows that sex is a critical determinant in the clinical manifestation and progression of MN, and that females are more resilient than males to renal injury following Rituximab therapy. This outcome will hopefully create new pathways to help identify the molecular pathways that underlie sex-related nephroprotective effects.

The goal of this Research Topic was also to provide pre-clinical contributions that addressed ways to halt the progression of renal injury, with a particular focus on diabetic nephropathy (DN). In this context, two studies describe different strategies to mitigate DN. Yi and colleagues show that erythropoietin, a glycoprotein hormone that is mainly secreted by the kidney, may have a beneficial effect during the development of DN by activating PINK1/Parkin-mediated mitophagy in DN mice.

Wei and collaborators demonstrate the effect that telmisartan, an angiotensin type 1 receptor inhibitor, has in alleviating diabetic kidney injury. By reducing podocyte injury and inhibiting mesangial cells proliferation, telmisartan prevented the early onset of renal abnormalities in streptozotocin-induced diabetes rats. Notably, the clinical study by Wu and colleagues included in the Research Topic shows that the use of angiotensin receptor blockers was associated with a lower risk of mortality after patients with dialysis-requiring acute kidney injury (AKI-D) were weaned off dialysis, while the use of angiotensin-converting enzyme inhibitors did not have beneficial effect on survival. Finally, a very interesting review by Borza and colleagues describes the role that discoidin domain receptors (DDRs), a family of transmembrane receptor tyrosine kinases (RTKs), play in kidney injury and disease. DDRs regulate several fundamental biological processes including cell adhesion, migration, proliferation, and extracellular remodeling. However, abnormal DDR expression has been documented in pathological conditions including cancer and inflammatory and fibrotic diseases. The review highlights canonical and non-canonical mechanisms through which DDRs contribute to kidney injury/fibrosis.

In conclusion, the Research Topic contributes to highlight how sex is a critical determinant in the clinical manifestation and progression of membranous nephropathy and also provides examples of recent advances in halting the progression of kidney damage.

